# Obstructive hydrocephalus due to posterior third ventricle pathology: One or two burr hole approaches for ETV and biopsy

**DOI:** 10.1007/s10143-026-04564-8

**Published:** 2026-11-06

**Authors:** Magomed Lepshokov, Sebastian Senger, Fritz Teping, Hannah Spielmann, Joachim Oertel

**Affiliations:** Department of Neurosurgery, Faculty of Medicine, Saarland University, Kirrberger Straße 100, Gebäude 90.5, 66424 Homburg/Saar, Germany

**Keywords:** Neuroendoscopy, Combined endoscopic procedures, Endoscopic third ventriculostomy, Endoscopic biopsy, Hydrocephalus, Intraventricular tumors

## Abstract

Endoscopic third ventriculostomy (ETV) and tumor biopsy is a frequent procedure for third ventricular tumors. While some authors favor one approach to achieve both goals, others support the selection of two approaches to avoid fornical damage and venous hemorrhage. Here the authors present their experience with both techniques. A retrospective analysis was conducted on cases which underwent an ETV and a tumor procedure in lesions of the posterior third ventricle from 2011 to 2025. Patient records, pre- and postoperative imaging and intraoperative video recordings were retrospectively analyzed. Twenty-six patients met the inclusion criteria: 13 were treated with one, the other 13 with two approaches. The surgical goal of ETV and tumor biopsy/resection was achieved in all cases. No procedure was abandoned. No postoperative complication was observed. Intraoperative forniceal injury was observed in 8/26 patients (31%). Intraoperative bleeding was found in 81% (21/26 cases). In 19% of cases (5/26 cases), intraoperative bleeding required a longer irrigation time but no switch to dry field technique or open surgery was needed. On follow up, three patients required ventriculoperitoneal shunt placement. Thus, ETV success scored 88.5% (23/26 cases). On 2D MRI, the mean intertrajectory angle was smaller in the one-burr-hole group than in the two-burr-hole group (41.8° ± 3.5° vs. 48.7° ± 5.4°; *p* = 0.001). Lesions of the posterior third ventricle causing obstructive hydrocephalus are ideal candidates for an endoscopic approach. The overall success rate is very high with acceptable surgical risks. In this cohort, larger intertrajectory angles were associated with selection of a two-burr-hole approach. The approximate 45° value was derived post hoc and requires external validation.

**Supplementary information:**

The online version contains supplementary material available at https://doi.org/10.1007/s10143-026-04564-8.

## Introduction

The posterior part of the third ventricle is one of the most difficult areas to access. The most frequent pathologies in this location are pineal tumors, tectal gliomas, metastases, germinomas and cysts [8, 22, 38]. The overall incidence of tumors in this region is 0.6% to 0.9% [3, 33]. The tumors frequently lead to an obstruction of the sylvian aqueduct resulting in an occlusive hydrocephalus in up to 90% of the cases [31, 49]. Frequently, surgical excision is not the first option in treating these lesions. Often endoscopic restoration of CSF circulation by ETV, and endoscopic biopsy of the lesion is the treatment strategy of first choice [2, 5, 11, 16, 27, 28, 30, 40, 41, 43].

ETV as the treatment of choice in these cases has been described in numerous publications [2, 4, 11, 16–18, 24, 26, 28, 30, 32, 35, 39, 41, 46, 48, 49]. Frequently the restoration of CSF circulation is combined with an endoscopic biopsy or endoscopic tumor resection. Rigid endoscopes or flexible endoscopes or a combination of both are currently used for combining CSF circulation restoration and tumor biopsy in the posterior third ventricle [1, 2, 6, 11, 12, 18, 21, 32, 39, 48].

Potential complications include forniceal injury, intraoperative bleeding, and, in rare cases, injury to critical vascular structures such as the basilar artery. While fornix injury might cause significant cognitive impairment, intraoperative bleeding might lead to a loss of intraoperative orientation. Particularly in free hand procedures without holding device and in procedure with the flexible endoscope, clearing the intraoperative surgical field with prolonged irrigation might be challenging due to the mobile nature of the techniques. Both surgical risks, fornix damage and venous hemorrhage, a highly depending on the individual anatomy of the foramen of Monro.

**Fig. 1:**
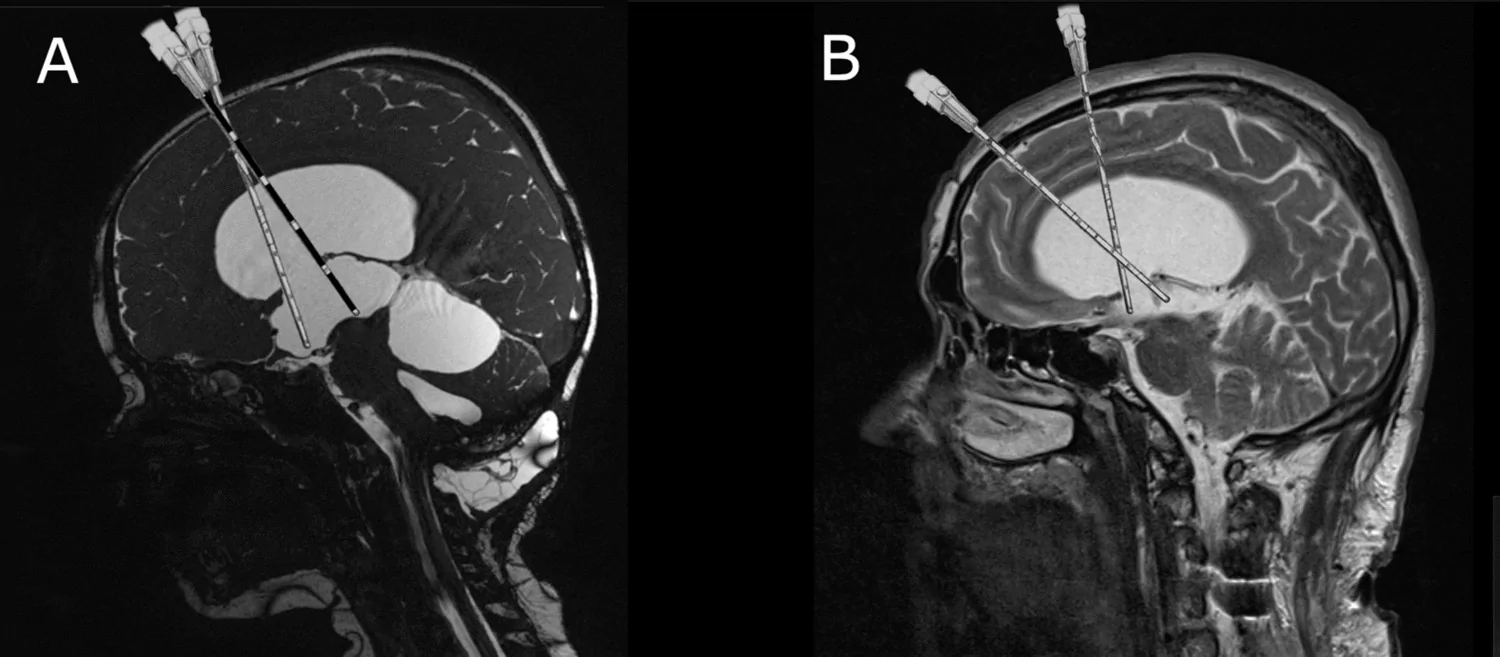
Schematic diagram of combined procedures using rigid endoscope. **A** – using one approach with rigid endoscope for ETV and approach to posterior part of the third ventricle; **B**—using two approaches with rigid endoscope for ETV and approach to posterior part of the third ventricle

There is an ongoing discussion in the neuroendoscopist community which technique should be preferred for these cases. While some neuroendoscopists favor a shear endoscopic approach through one burr hole with a rigid endoscope, others favor a combination of a rigid endoscope for the ETV and a flexible endoscope for the tumor procedure. Others again recommend two separate straight approach to the floor of the third ventricle and to the posterior part of the third ventricle (Fig. 1) [31]. In particular, the angle between endoscopic third ventriculostomy and tumor biopsy trajectories, ventricular geometry, and anatomical constraints such as the massa intermedia critically influence procedural feasibility, ergonomics, and the volume of brain at risk [16, 25].

Here the authors analyzed their series of combined endoscopic procedures for posterior third ventricle lesions and ETV retrospectively for the different approaches and trajectories, individual intraventricular anatomy, intraoperative complications and overall success rate.

## Materials and methods

All intraventricular procedures performed in the Department of Neurosurgery, Saarland University Medical Center, Homburg, Germany were analyzed retrospectively from 2011 till 2025. Inclusion criteria were complete patient records, pre- and postoperative imaging, intra- and postoperative complications and intraoperative video recordings. Patients with incomplete pre- and postoperative imaging, missing intraoperative video, or insufficient follow-up were excluded.

### Surgical technique

All procedures were performed under of general anesthesia in a supine position with rigid endoscopes. The head was slightly anteflexed and fixed in a Mayfield clamp or in selected cases with tight bandages. All approaches were paramedian standard approaches with a burr hole positioned 2–3 cm lateral from midline.

One day preoperatively, detailed surgical planning was performed in all cases. The choice between a one- and two-burr-hole approach was made preoperatively on a case-by-case basis. No predefined numerical angle threshold or formal decision algorithm was used. The decision was based on neuronavigation planning, individual ventricular anatomy, anticipated divergence of the trajectories for ETV and tumor biopsy/resection, estimated risk of forniceal manipulation, procedural feasibility, and the surgeon’s experience. The vast majority of procedures in both groups were planned and performed by the same senior neurosurgeon with extensive experience in neuroendoscopic surgery.

In combined procedures, ETV was performed first, followed by biopsy, fenestration, or resection of the lesion, as indicated. For tumor biopsies, tissue samples were obtained before coagulation. When bleeding occurred, irrigation and, when necessary, bipolar coagulation were used for hemostasis.

For one single approach, a straight trajectory from the optimum burr hole position for ETV through the foramen of Monro to the floor of the third ventricle was calculated on one hand, a second straight trajectory from the optimum burr hole position for the tumor procedure through the foramen of Monro to the third ventricle tumor was calculated on the other hand. The entry point was defined in between the two ideal burr hole positions calculated by neuronavigation.

For two approaches, ideal trajectories were calculated as described for one single approach. However, the two distinct ideal burr hole positions for ETV and the tumor procedure were used for the separate straight approaches. For the rostral approach, the ideal burr hole position frequently was far anterior. Thus, either a longer skin incision just behind the hair line with subsequent mobilization of the skin anteriorly, or if indicated, a small skin incision on the forehead was performed.

### Data analysis

#### Qualitative descriptive analysis

The procedures were analyzed regarding type of approaches (one burr hole or two burr holes), surgical time, intraoperative bleeding and damage of the fornix. Postoperative and follow-up CT/MRI scans were analyzed for postoperative hemorrhages and progression of hydrocephalus. Postoperative cognitive status was assessed from routine clinical neurological evaluations and available documentation. No standardized cognitive assessment was performed pre- or postoperatively.

#### Semiquantitative analysis

Particular focus was given to fornical damage and intraoperative hemorrhages. Additionally to the qualitative analysis, an earlier published grading system for intraoperative bleeding and fornical damage was applied [10]. The ependymal damage was assessed as Grade 0 (no lesion), Grade I (contusion with maintained surface integrity), Grade II (contusion with intact ependymal but distorted surface) and Grade III (loss of ependymal integrity and anatomic structural loss).

The intraoperative hemorrhages were assessed as Grade I (small streak bleeding without visual impairment), Grade II (hemorrhage with blurry vision but remained anatomical orientation), Grade III (hemorrhage with blurry vision and lost identification of anatomical landmarks) and Grade IV (complete loss of vision, “red out”).

#### Retrospective analysis of anatomy and approaches

All radiological data were evaluated for:

- height, width and area of the foramen of Monro;
- intertrajectory angle—the angle formed between the trajectories for an ideal ETV and the trajectory for an ideal tumor procedure;
- distance from the posterior edge of the foramen of Monro to the lesion;
- the angle formed by the lateral and third ventricle.

**Fig. 2:**
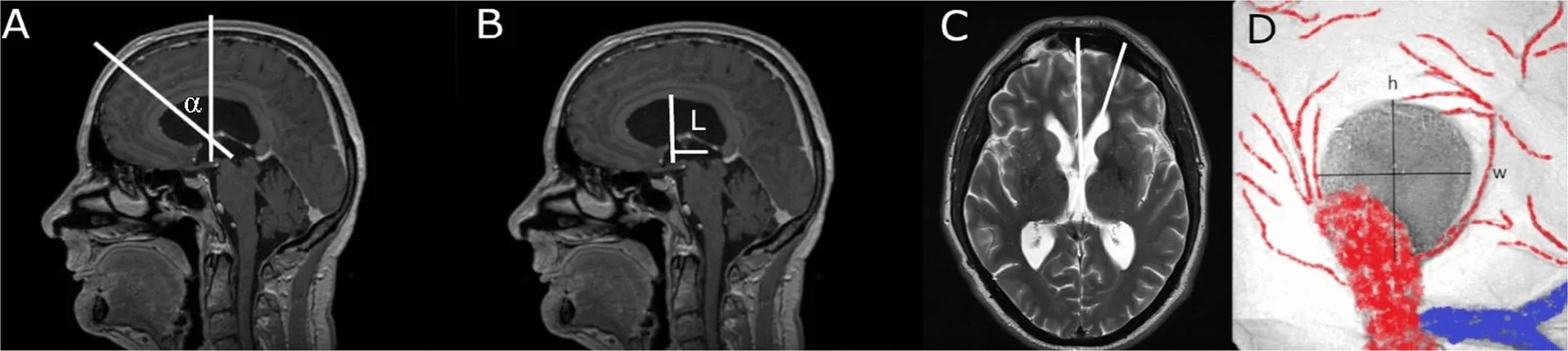
**A**—intertrajectory angle (α) (defined as the angle between the ideal trajectory to the floor of the third ventricle for ETV and the ideal trajectory to the posterior third ventricular lesion for biopsy or resection); **B**—distance between foramen of Monro and lesion; **C**—angle between lateral and third ventricle; **D** – measurement of foramen of Monro (h – height, w – width)

The various measurements are displayed in Fig. 2.

**Fig. 3:**
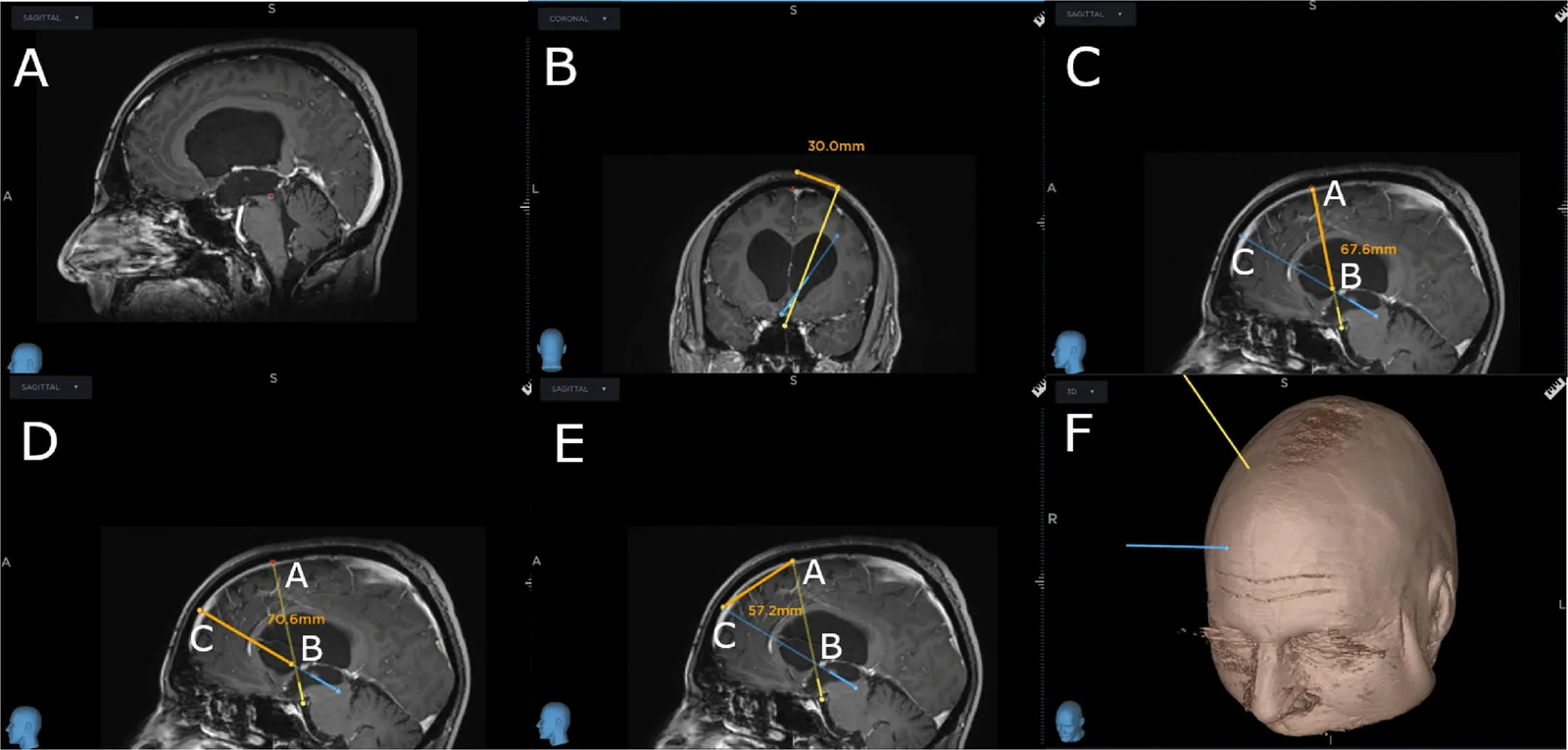
**A** – Tumor in the area of the Sylvian aqueduct (red dot); **B** – Planning of the optimal access point for ETV in the coronal plane (yellow line); **C** – Planning of ideal trajectories to the Sylvian aqueduct (blue line) and measurement of distance *AB*; **D** – Measurement of distance *BC*; **E** – Measurement of distance *AC* and determination of angle *ABC* using the formula for calculating angles in a scalene triangle; **F** – 3D model showing the direction of ideal trajectories

To address differences between 2-D preop images and 3-D surgical planning navigational images, all data were again imported into the navigational unit. Subsequently, differences between ideal trajectories on two-dimensional MRI images in the sagittal plane and the 3D-model on the navigation planning station were assessed (Medtronic Ltd.) (Fig. 3).

#### Statistical evaluation

Statistical analysis was performed using SPSS 29 (SPSS Inc., Chicago, Illinois). Descriptive statistics were used to summarize the characteristics of both groups. Continuous variables were compared between the one- and two-burr-hole groups using the independent-samples Student’s t-test, while paired 2D MRI and 3D navigation measurements were compared using the paired Student’s t-test. Categorical variables were compared using Fisher’s exact test. All reported p-values were two-sided, with statistical significance set at *p* < 0.05. Unadjusted odds ratios (ORs) comparing the two-burr-hole with the one-burr-hole group and approximate 95% confidence intervals (CIs) were calculated for forniceal injury and intraoperative bleeding. The retrospective study was approved by the Saarland Ethics Committee.

## Results

Of the 2,425 endoscopic procedures performed during the study period, 165 involved ETV combined with an additional intraventricular intervention (biopsy or resection). Among these, 26 cases met the inclusion criteria, representing 15.8% of all combined procedures and 1.07% of all endoscopic procedures. These cases included patients with tumors or cysts of the posterior third ventricle. The sample consisted of 26 patients (9 men and 17 women, mean age 41 years, range 1 year to 73 years). Tumor lesions were diagnosed in 19 patients, cysts in 7 patients. For a detailed compilation of the cases please refer to Table 1.

**Table 1:** Summary of the combined procedures

Patient	Age	Sex	Location	Histology	Procedere	Number of approaches
1	45	w	Third ventricel	Pilocytic Astrocytoma WHO I	Resection + ETV	1
2	55	m	Periaqueductal	Pilocytic Astrocytoma WHO I	Biopsy + ETV	1
3	50	w	Periaqueductal	Pilocytic Astrocytoma WHO I	Biopsy + ETV	1
4	61	w	Third ventricel	Cyst	Fenestration + ETV	1
5	15	m	Pineal region	Pineal cyst	Resection + ETV	1
6	11	w	Tectal region	Pilocytic Astrocytoma WHO Grad I	Resection + ETV	1
7	12	w	Periaqueductal	Low-grade astrocytoma WHO Grad II	Biopsy + ETV	1
8	10	w	Periaqueductal	Pilocytic Astrocytoma WHO Grade I	Biopsy + ETV	1
9	14	w	Periaqueductal	Pilocytic Astrocytoma WHO Grade I	Biopsy + ETV	1
10	66	w	Periaqueductal	Low-grade astrocytoma WHO Grad II	Biopsy + ETV	1
11	31	w	Pineal region	Pineal cyst	Fenestration + ETV	1
12	12	m	Periaqueductal	Germinom	Biopsy + ETV	1
13	40	w	Periaqueductal	Pilocytic Astrocytoma WHO Grade I	Biopsy + ETV	1
14	73	w	Pineal region	Anaplastic astrocytoma WHO Grad III	Resection + ETV	2
15	70	w	Periaqueductal	Metastatic small cell carcinoma	Biopsy + ETV	2
16	58	w	Pineal region	Glioblastoma WHO Grad IV	Biopsy + ETV	2
17	14	w	Pineal region	Pineal cyst	Fenestration + ETV	2
18	58	w	Third ventricel	Colloid cyst	Resection + ETV	2
19	46	m	Pineal region	Pineal cyst	Fenestration + ETV	2
20	16	m	Tectal region	Pilocytic Astrocytoma WHO Grad I	Biopsy + ETV	2
21	1	w	Tectal region	Cyst	Fenestration + ETV	2
22	48	m	Periaqueductal	Low-grade astrocytoma WHO II	Biopsy + ETV	2
23	62	m	Third ventricel	Metastatic carcinoma	Biopsy + ETV	2
24	62	m	Pineal Region	Metastatic carcinoma	Biopsy + ETV	2
25	73	m	Pineal Region	Pineal parenchymal tumor of intermediate differentiation	Biopsy + ETV	2
26	70	w	Periaqueductal	Metastatic carcinoma	Biopsy + ETV	2

All patients suffered from obstructive hydrocephalus due to compression or occlusion of the Sylvian aqueduct. The main presenting symptoms were headache in 11 patients (42%), gait difficulties in 9 patients (34%), vomiting in 4 patient (15%), delirium in 3 patient (11%), diplopia in 3 patient (11%). Focal neurological symptoms were extremely rare, as paresis were noted in 1 patient (4%).

Surgical treatment through one approach was performed in 13 patients (50%) and through two burr holes in the other 13 patients (50%).

### Case example

#### One approach

**Fig. 4:**
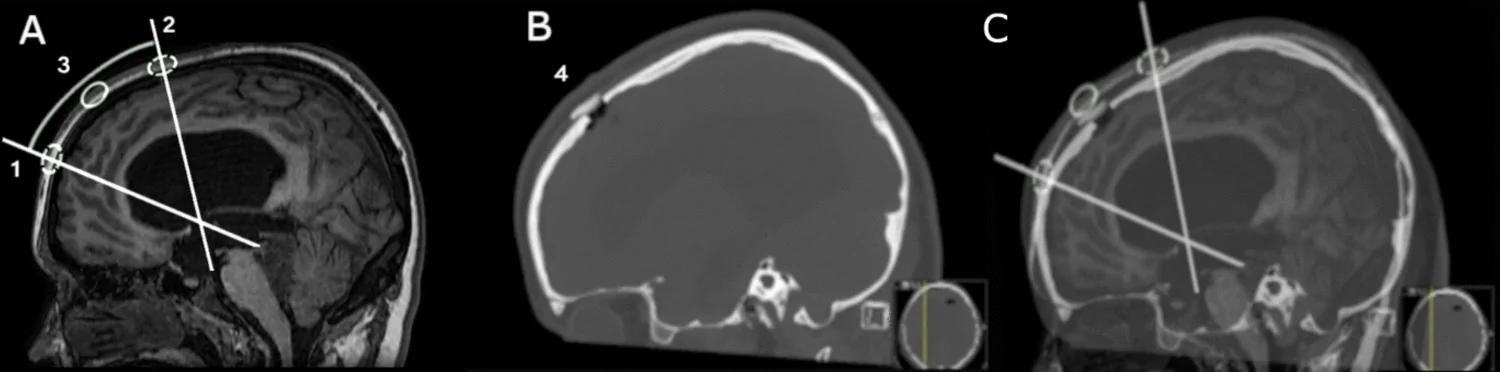
**A** – preoperative planning on sagittal MRI:1—ideal trajectory to the tumor for biopsy/resection, 2 – ideal trajectory for endoscopic third ventriculostomy (ETV). Dashed ovals indicate the burr hole projections corresponding to each ideal trajectory. 3 – solid oval represents the projection of a single burr hole placed at the midpoint between the two ideal trajectories, enabling both procedures through one access point. **B** – postoperative CT scan of the patient who underwent tumor biopsy and ETV through a single burr hole, 4—burr hole location for single approach. **C** – fusion of preoperative planning with postoperative CT, demonstrating alignment of the planned trajectories and burr hole with the single approach used for both biopsy and ETV

A 14 years old girl presented to our hospital with a 3-week history of headache. The neurological examination showed no focal neurological symptoms. Magnetic resonance imaging of the brain revealed a tumor mass, with obstruction of the sylvian aqueduct and associated obstructive hydrocephalus (Fig. 4). After the planning on the neuronavigation station, the decision was made to approach the lesion and perform the ETV via one approach with the burrhole in the middle between the ideal straight anterior and the ideal straight posterior trajectory. Through this right frontal burr hole, the procedure was performed endoscopically with ETV and biopsy (video 1). The biopsy results revealed pilocytic astrocytoma.

#### Two approaches

**Fig. 5:**
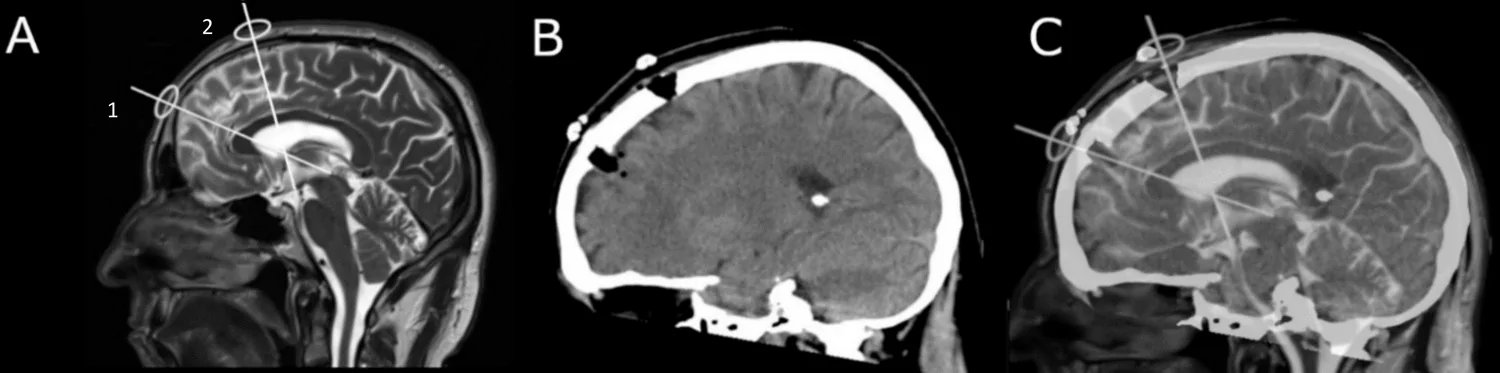
Preoperative and postoperative view of a 46 old male with pineal cyst. **A** – preoperative planning on sagittal MRI:1—ideal trajectory to the cyst for resection, 2 – ideal trajectory for endoscopic third ventriculostomy (ETV) (solid oval represents the projection of a burr holes), **B** – postoperative CT scan of the patient who underwent cyst resection and ETV through a two burr holes. **C** – fusion of preoperative planning with postoperative CT, demonstrating alignment of the planned trajectories and burr holes used for both cyst resection and ETV

A 46-year-old male was admitted with severe headache, nausea, and vomiting. MRI revealed a cystic lesion in the pineal region with compression of the Sylvian aqueduct and resulting obstructive hydrocephalus. Following preoperative planning with neuronavigation, it was decided to perform ETV and cyst fenestration through two separate burr holes (video 2). For cyst fenestration, the optimal trajectory was planned through the center of the foramen of Monro to pineal region, and a burr hole was placed rostrally in the frontal region. The burr hole for ETV was placed caudally, in alignment with the ideal trajectory passing through the center of the foramen of Monro toward the floor of the third ventricle (Fig. 5). Histopathological examination confirmed a benign cyst. Postoperatively, symptoms improved, and no recurrence was observed during follow-up.

#### Analysis of the procedures and complications

The duration of surgery ranged from 28 to 122 min (mean 66.2 min). In the one single approach group, the mean duration was 59.3 min, while in the two-approach group it was 73.1 min (t-test = − 1.39, *p* = 0.177). In all cases, ETV and biopsy/resection were completed as planned. No conversion to an alternative access strategy or additional burr hole was required. ETV success, defined as the absence of subsequent VP-shunt placement, was achieved in 23 of 26 patients (88.5%); three patients (11.5%) subsequently required VP-shunt placement. Histopathological diagnosis was confirmed in all cases.

Superficial fornix injuries (Grade I) were observed in 7 patients (27%), and moderate injuries (Grade II) in 1 patient (4%). Fornix damage was more frequent in the two-approach group (46.2%) compared to the one-approach group (15.4%), though the difference was not statistically significant (Fisher’s exact test, *p* = 0.202) (Table 2). Intraoperative bleeding occurred in 21 patients (81%). In 16 patients (62%), bleeding was classified as Grade I or II, with anatomical orientation remaining preserved. Grade III or IV bleeding occurred in five patients (19.2%). Bleeding was more frequent in the two-approach group (92.3%) compared to the one single approach group (69.2%), though the difference was not statistically significant (Fisher’s exact test, *p* = 0.322) (Table 2). In all cases, bleeding was effectively controlled using continuous irrigation and bipolar coagulation. With routine postoperative clinical evaluation, no new overt cognitive deficits were documented. As standardized testing was not available, subtle cognitive changes and an association between forniceal injury grade and cognitive outcome could not be assessed. No postoperative complications requiring surgical intervention were observed.

**Table 2:** Comparison of anatomical, procedural, outcome parameters and intraoperative findings between the one- and two-burr-hole groups

Parameter	One-burr-hole approach (*n* = 13)	Two-burr-hole approach (*n* = 13)	*p*-value
Anatomical parameters			
Intertrajectory angle on 2D MRI, degrees	41,8° ± 3,5	48,7° ± 5,4	0.001
Intertrajectory angle on 3D navigation, degrees	42,0° ± 6,15	51,0° ± 3,39	< 0.001
Angle between the lateral and third ventricles, degrees	19,05° ± 6,32	19,85° ± 3,9	0.704
Distance from the posterior edge of the foramen of Monro to the lesion, mm	17,14 ± 8,53 mm	18,8 ± 4,16 mm	0.532
Height of the foramen of Monro, mm	7,16 ± 2,84 mm	6,39 ± 3,14 mm	0.518
Width of the foramen of Monro, mm	7,4 ± 3,62 mm	7,1 ± 3,9 mm	0.845
Area of the foramen of Monro, mm^2^	189,7 ± 139,44 mm^2^	172,85 ± 178,6 mm^2^	0.791
Procedural parameters			
Operative time, min	59.3	73.1	0.177
Successful completion of ETV and biopsy/resection	13 (100%)	13 (100%)	1.000
Histopathological diagnosis obtained	13 (100%)	13 (100%)	1.000
Intraoperative findings			
Any forniceal injury*	2 (15.4%)	6 (46.2%)	0.202
Forniceal injury Grade 0	11	7	—
Forniceal injury Grade I	2	5	—
Forniceal injury Grade II	0	1	—
Forniceal injury Grade III	0	0	—
Any intraoperative bleeding*	9 (69.2%)	12 (92.3%)	0.322
Bleeding Grade I	4	6	—
Bleeding Grade II	3	3	—
Bleeding Grade III	1	1	—
Bleeding Grade IV	1	2	—
ETV success (no subsequent VP shunt)	12 (92.3%)	11 (84.6%)	1.000

^*^: Unadjusted odds ratios (ORs) comparing the two-burr-hole with the one-burr-hole approach were 4.71 (95% CI 0.73–30.28) for forniceal injury and 5.33 (95% CI 0.51–56.24) for intraoperative bleeding. P-values were calculated using Fisher’s exact test. Confidence intervals were estimated using the Wald method

#### Follow-up and clinical outcomes

All 26 patients were available for early postoperative follow-up after hospital discharge. At 6-month follow-up, one patient had died. Among the remaining 25 patients, functional outcome according to the modified Rankin Scale (mRS) was mRS 0 in 19 patients (76%), mRS 1 in 3 patients (12%), and mRS 2 in 3 patients (12%). At 12-month follow-up, two additional deaths were recorded. Among the remaining 23 patients, outcomes were mRS 0 in 19 patients (82%), mRS 1 in 2 patients (9%), and mRS 2 in 2 patients (9%). Overall, three patients required subsequent VP-shunt placement, including two patients with tumor-related progression of hydrocephalus.

#### Retrospective analysis of radiological anatomy and in the context of approaches

Comparison of the distance from the posterior edge of the foramen of Monro to the lesion, as well as the height, width, and area of the foramen, and the angle between the lateral and third ventricles, revealed no significant differences between the one-approach and two-approach groups (*p* > 0.05) (Table 2).

The intertrajectory angle was the only anatomical parameter that differed significantly between the groups. On 2D MRI planning, the mean angle was 41.8° ± 3.5° in the one-burr-hole group and 48.7° ± 5.4° in the two-burr-hole group (*t* = − 3.84, *p* = 0.001). Measurements obtained from the 3D neuronavigation planning models confirmed this difference, with mean angles of 42.0° ± 6.15° in the one-burr-hole group and 51.0° ± 3.39° in the two-burr-hole group (*t* = − 4.6, *p* < 0.001).

The measurements obtained from 2D MRI and 3D neuronavigation planning were compared for each patient. No statistically significant difference was found between the two measurement methods (*t* = − 1.25, *p* = 0.224).

#### Criteria for one or two approaches

Based on the conducted analysis, we recommend the use of preoperative MRI—specifically T1 MP-RAGE or T2 TRUFI sequences—for access planning. Multiplanar reconstruction (MPR) is used to define the ideal trajectories for biopsy/resection and ETV in the sagittal plane.

Retrospective analysis demonstrated that larger angles between the ideal trajectories for ETV and tumor biopsy/resection were more frequently associated with the use of a two-burr-hole approach. The approximate value of 45° was derived post hoc from the observed mean angle plus one standard deviation and was not used for surgical decision-making. It should therefore be regarded as a descriptive, hypothesis-generating observation derived from the present cohort rather than as a prescriptive or clinically validated threshold. External validation in an independent cohort is required before this value can be considered for clinical decision-making.

## Discussion

The choice of surgical access in combined neuroendoscopic procedures remains disputable [14, 23–25, 34, 39, 45, 50].

There are various variations in planning of the approach: one burr hole performed between two ideal trajectories for ETV and biopsy [1, 2, 24, 28, 34, 47]; using two burr holes, one for ETV and other for biopsy [6, 10, 11, 15, 21, 23, 45, 50]; one burr hole using a flexible endoscope [2, 19, 34, 41]; one burr hole and using a combination of flexible and rigid endoscope [12, 28, 35, 38, 39]. The computer assisted methods are also used to identification an ideal access point [24]. A summary of the described techniques is provided in Table 3.

**Table 3:** Review of combined surgical approaches for lesions in the posterior third ventricular region

No	Author/Year	No. of patients/No. of patients with combined procedure	Approach Type	Surgical technique	Histopath. Dx. Achieved (%)	ETV Success	Histopath. Dx (n)	Complications (n)	Location (n)
1	Veto, F.Z., et al., 1997 [45]	3/2	two approaches (rigid endoscope)	ETV + endoscopic biopsy	100%	100%	metastatic carcinoma (1) cavernoma (1) malignant tumor (1)	no	posterior third ventricle (3)
2	Javedan S.P., et al., 2003 [21]	1/1	two approaches (rigid endoscope)	ETV + endoscopic biopsy	100%	100%	central neurocytoma (1)	no	posterior third ventricle (1)
3	Kim, I.Y., et al., 2004 [23]	5/5	two approaches (rigid endoscope)	ETV + endoscopic biopsy	100%	100%	pineoblastoma (1) germinoma (2) pineocytoma (1) embryonal cell carcinoma (1)	no	pineal region (5)
4	O’Brien, D.F., et al., 2006 [28]	42/33	one approach (combined rigid-flexible endoscopic technique)	ETV + endoscopic biopsy	76%	68%	no information	transient 6th cranial nerve paresis (2) intraoperative hemorrhage (1) intratumoral hemorrhage (1) (*group affiliation unclear from the article*)	midbrain (3), pons (1), thalamus (3), tectal plate (9) pineal region (24), third ventricle (2)
5	Boscherini D., et al., 2006 [6]	1/1	two approaches (rigid endoscope)	ETV + endoscopic biopsy	100%	100%	metastatic adenocarcinoma (1)	no	pineal region (1)
6	Chernov, M.F., et al., 2006 [10]	23/23	two approaches (rigid endoscope)	ETV + endoscopic biopsy	100%	96%	germinoma (8), glioma (8), neuronal tumor (2), lymphoma (1), metastasis (1), epidermoid (1), nonneoplastic lesions (2)	transient fever (15) transient diplopia (1)	posterior third ventricle, pineal region
7	Shono, T., et al., 2007 [41]	12/8	one approach (flexible endoscope)	ETV + endoscopic biopsy	100%	100%	germinoma (12)	transient fever (5); transient hemiparesis (1)	posterior third ventricle, pineal region
8	Costa, F., et al., 2008 [15]	1/1	two approaches (rigid endoscope)	ETV + cyst fenestration	100%	100%	pineal cyst (1)	no	pineal region (1)
9	Al-Tamimi, Y.Z., et al., 2008 [2]	12/8	one approach (rigid endoscope or flexibel endoscope)	ETV + endoscopic biopsy	75%	88%	germinoma (3), pinealoblastoma (1), astrocytoma (1), mature teratoma (1)	transient worsening of diplopia (2)	pineal region (12)
10	Cinalli, G., et al., 2010 [12]	14/2	one approach (combined rigid-flexible endoscopic technique)	ETV + cyst fenestration	100%	100%	arachnoid cyst (14)	intraoperative bleeding (1)	quadrigeminal cistern (14)
11	Oppido, P.A., et al., 2011 [34] multicenter study (7 centers)	60/32	one approach (rigid endoscope and flexibel endoscope)	ETV + endoscopic biopsy	90%	94%	low-grade glioma (7), high-grade glioma (9), pure germinoma (9), malignant teratoma (1), pinealoblastoma (2), pineocytoma (5), craniopharyngioma-(4), primitive neuroectodermal tumor (5), lymphoma (6), metastasis (3), nonspecific tumor (5), inconclusive (6)	IVH (8), epilepsy (2), hydrocephalus (1), meningitis (1), transient Parinaud syndrome (1), intracerebral hematoma subsequent to ventricular puncture (1) (*group affiliation unclear from the article*)	pineal region (23), thalamic region (12), mesencephalon (11)
12	Chibbaro, S., et al., 2012 [11]	20/20	two approaches (rigid endoscope)	ETV + tumor resection (12); ETV + endoscopic biopsy (8)	100%	100%	germinoma (8), glioblastoma (1), epidermoid cyst (3), neurocytoma (2), pilocytic astrocytoma (2), ependymoma (1), pinecytoma (1); benign pineal cyst (1), primitive neuroectodermal tumor (1)	bilateral ophthalmoplegia (1), intratumoral hematoma (1), vegetative state (1)	posterior third ventricle, pineal region
13	Roth, J., et al., 2015 [38] multicenter study (3 centers)	16/2	one approach (combined rigid-flexible endoscopic technique)	ETV + endoscopic biopsy	100%	100%	glioneural tumor (1), one non-specified low- grade glioma (1)	non information	Sylvian aqueduct (2)
14	Roth, J., et al., 2015 [39]	6/6	one approach (combined rigid-flexible endoscopic technique)	ETV + endoscopic biopsy	100%	83%	low-grade glioneural tumor – 2, pinealoblastoma – 1, juvenile pilocystic astrocytoma – 1, germinoma – 1, atypical choroid plexus papilloma – 1	IVH (1)	4th ventricle (1), pineal (3), aqueduct (1), tectal (1)
15	Abbassy, M., et al., 2018 [1]	11/11	one approach (rigid endoscope)		81,8%	100%	germ-cell tumors (3), pinealoblastoma (1), pilocytic astrocytomas (2), tectal gliomas (2)	IVH (1), tumor dissemination (1)	pineal region (11)

Neuronavigation was used in all cases to define the ideal trajectories and support intraoperative orientation. The use of neuronavigation in planning surgical access allows reducing the risks of complications [23, 36, 37]. Among the anatomical parameters analyzed, the intertrajectory angle was the only variable that differed significantly between the one- and two-burr-hole groups, with larger angles observed in the two-burr-hole group. This angle reflects the divergence between the optimal trajectory to the floor of the third ventricle for ETV and the trajectory to the posterior third ventricular lesion for biopsy or resection. Greater divergence may require increased endoscope angulation and manipulation at the foramen of Monro when a single compromise trajectory is used. As anticipated trajectory divergence was considered during preoperative approach selection, the observed association between the angle and the chosen technique may partly reflect this original decision-making process. The approximate value of 45° was derived post hoc from the mean angle in the one-burr-hole group plus one standard deviation (41.8° + 3.5° = 45.3°). It should therefore be regarded as a descriptive, hypothesis-generating observation rather than as a clinically validated decision threshold and requires external validation. As no statistically significant difference was found between measurements obtained from 2D MRI and 3D neuronavigation models, MRI-based assessment may provide a practical method for preoperative evaluation of the intertrajectory angle when neuronavigation is unavailable. A prominent massa intermedia may restrict endoscopic visualization and instrument maneuverability, particularly when the lesion is located posterior to it [5, 26]. However, this parameter was not systematically measured in our cohort, and its influence on the choice of surgical approach could therefore not be evaluated.

It is not always possible to determine the histological type of tumor in neuroendoscopic biopsy. The frequency reaches in the literature 75%—100% [2, 10, 11, 30]. There is no direct dependence on the type of endoscope or the number of approaches [2]. In our study, it was possible to histologically verify the type of tumor in all patients.

Intraoperative bleeding are the most potential dangerous and frequent event during endoscopic biopsy [10, 13, 14]. They bare the risk of an instant loss of vision and that could cause major problems. The rate of intraoperative bleeding during biopsy was 75% according to a large series of Constantini et al. [14]. The frequency of intraoperative bleeding in ETV is several times lower. So according to Bouras et al. frequency is 3,72% [7]. For severe intraoperative hemorrhage that cannot be controlled by conventional irrigation and coagulation, the “dry-field technique” has been described as an effective method of achieving hemostasis [29]. Tumors are usually well-supplied and an extremely high risk of hemorrhagic complications is associated with this. In our series, intraoperative bleeding occurred in 81% of patients. However, most bleeding events were Grade I or II (16/21, 76%), with preserved anatomical orientation. All bleeding events were managed endoscopically without conversion to open surgery. There was not severe bleeding, which required EVD implantation. No statistically significant difference in intraoperative bleeding rates was observed between the one- and two-burr-hole groups. Postoperative hemorrhagic complications have been reported in 2–19% of cases [14, 20], mainly as intraventricular hemorrhage or parenchymal hemorrhage along the endoscopic tract. In our series, postoperative CT imaging revealed only minor blood traces along the endoscopic trajectory, without clinically significant postoperative hemorrhage. The frequency of forniceal injury reported in the literature ranges from 0.24% to 16.4% [7, 30, 46]. Most injuries are asymptomatic, with persistent memory deficits reported in only 0.17% of cases [7, 30, 42]. In our series, Grade II forniceal contusions occurred in 4% of patients. No overt cognitive deficits were identified during routine postoperative clinical evaluation. However, in the absence of formal neuropsychological testing, subtle cognitive impairments and their association with forniceal injury could not be reliably assessed. Although higher rates of forniceal injury and intraoperative bleeding were observed in the two-burr-hole group, these differences did not reach statistical significance. The findings should be interpreted cautiously because of the small sample size and potential selection bias toward anatomically more challenging cases in the two-burr-hole group. Therefore, the absence of statistically significant differences should not be interpreted as evidence of equivalence between the two approaches.

Prolonged operative time increases the risk of infectious complications [9, 44, 51]. This is especially important in surgical interventions on the ventricular system [44]. There were no infectious complications in our series. In this case, it should be taken into account that the size of the cutaneous incision and the size of craniotomy were minimal. At the same time, performing the operation from one approach can be technical difficulties and lengthen the intervention.

One of the main issues in combined endoscopic procedures is the use of a flexible endoscope. In our opinion, the rigid endoscope appears to be a reliable option for biopsy in the posterior third ventricle. In selected cases with markedly divergent trajectories, an additional approach may provide more direct surgical corridors and facilitate endoscopic instrument handling. However, the present study was not sufficiently powered to determine equivalence in complication rates between the two strategies. The use of a flexible endoscope is an excellent option for providence inspection with evaluation of tumor altered normal ventricular system anatomy and for biopsy planning or stenting. Flexible endoscopes are limited due to their lower resolution of image quality and poor possibility for managing of the bleeding. Therefore experience and knowledge of the individual anatomy is mandatory. Also the manoeuvrability can be limited when the instruments are inserted and the region of interest may not be reached for manipulation.

## Conclusion

Lesions of the posterior parts of the third ventricle and pineal region with concomitant hydrocephalus are a very rare pathology. The endoscopic technique carries minimal risk for the patient, shortens the duration of the operation, and in most cases eliminates the need for complex procedures. In our experience, the rigid endoscope appears to be a reliable tool for biopsy in the posterior parts of the third ventricle.

The anatomy of the third ventricle and the relationship between the foramen of Monro, the “loco typico” for ETV, and the lesion itself is crucial for planning combined endoscopic procedures. Preoperative planning of the necessary number of endoscopic approaches based on MRI scans is a simple and effective method, especially valuable in the absence of neuronavigation systems.

In this cohort, no statistically significant increase in complication rates was detected with the two-burr-hole approach; however, the limited sample size precludes conclusions regarding equivalence between the two strategies. In selected cases with markedly divergent trajectories, the dual-approach technique may provide more direct surgical corridors and facilitate endoscopic instrument handling.

## Limitations

This study is limited by its retrospective single-center design and small sample size, which reduce statistical power. Therefore, the absence of significant differences in complication rates should not be interpreted as evidence of equivalence between the two approaches. Formal neuropsychological assessment, including age-appropriate testing in children, was not performed. Therefore, subtle cognitive deficits and their potential association with forniceal injury could not be reliably assessed. The choice of approach was made individually rather than according to predefined numerical criteria, introducing a potential selection bias toward the use of two burr holes in anatomically more challenging cases. The approximate 45° value was derived post hoc and requires external validation. Although most procedures were performed by the same experienced neuroendoscopic surgeon, this may limit the generalizability of our findings.

## Supplementary information

Below is the link to the electronic supplementary material.

Supplementary file1 (MP4 1830 KB)

Supplementary file2 (MP4 1830 KB)

## Data Availability

Raw data are available in an anonymous fashion upon request.
